# Neuroleptic-induced movement disorders in a naturalistic schizophrenia population: diagnostic value of actometric movement patterns

**DOI:** 10.1186/1471-2377-8-10

**Published:** 2008-04-18

**Authors:** Sven Janno, Matti M Holi, Katinka Tuisku, Kristian Wahlbeck

**Affiliations:** 1 Department of Psychiatry, University of Tartu, Raja 31, 50417, Tartu, Estonia; 2 Department of Psychiatry, University of Helsinki, Tukholmankatu 8 C, 00029, Finland; 3 Kellokoski Hospital, Hospital District of Helsinki and Uusimaa, FIN-04500 Kellokoski, Finland; 4 Mehiläinen Ltd, Siltasaarenkatu 18A, FIN-00530 Helsinki, Finland; 5 STAKES National Research and Development Centre for Welfare and Health, P.O.Box 220, FIN-00531 Helsinki, Finland; 6 Psychiatric Unit, Vaasa Central Hospital, Hietalahdenkatu 2-4, FIN-65130 Vaasa, Finland

## Abstract

**Background:**

Neuroleptic-induced movement disorders (NIMDs) have overlapping co-morbidity. Earlier studies have described typical clinical movement patterns for individual NIMDs. This study aimed to identify specific movement patterns for each individual NIMD using actometry.

**Methods:**

A naturalistic population of 99 schizophrenia inpatients using conventional antipsychotics and clozapine was evaluated. Subjects with NIMDs were categorized using the criteria for NIMD found in the Diagnostic and Statistical Manual for Mental Disorders – Fourth Edition (DSM-IV).

Two blinded raters evaluated the actometric-controlled rest activity data for activity periods, rhythmical activity, frequencies, and highest acceleration peaks. A simple subjective question was formulated to test patient-based evaluation of NIMD.

**Results:**

The patterns of neuroleptic-induced akathisia (NIA) and pseudoakathisia (PsA) were identifiable in actometry with excellent inter-rater reliability. The answers to the subjective question about troubles with movements distinguished NIA patients from other patients rather well. Also actometry had rather good screening performances in distinguishing akathisia from other NIMD. Actometry was not able to reliably detect patterns of neuroleptic-induced parkinsonism and tardive dyskinesia.

**Conclusion:**

The present study showed that pooled NIA and PsA patients had a different pattern in lower limb descriptive actometry than other patients in a non-selected sample. Careful questioning of patients is a useful method of diagnosing NIA in a clinical setting.

## Background

Globally, many schizophrenia patients still suffer from neuroleptic-induced movement disorders (NIMDs) [1-3] in spite of the increasing use of second generation antipsychotics, which in general have a lower propensity for NIMDs [4]. Although in earlier years movement adverse effects were even considered to be an indicator of antipsychotic action [5], today they are seen as burdening and stigmatizing phenomena that should be avoided by careful choice of treatment [6]. NIMDs, which are investigated in this study and classified in DSM-IV [7], include neuroleptic-induced akathisia (NIA), neuroleptic-induced parkinsonism (NIP) and tardive dyskinesia (TD). Pseudoakathisia (PsA) is a movement disorder with objective signs of akathisia, but without the subjective feeling of restlessness [8]. PsA is not included in the DSM-IV classification [7].

Besides motor inconvenience, NIMD can cause subjective suffering through cognitive or emotional disturbance [9]. Patients have described rigidity and dullness of thinking in association with parkinsonism [10]. Akathisia causes significant distress and is reported to be associated with suicide attempts [11] and aggression [12]. It results in reduced compliance with antipsychotic drug treatment and thus a cause of psychotic relapses [13].

The interest in the instrumental measurement of NIMDs in addition to clinical examination and observer-based rating scales has long traditions. Instrumental measurement has been reported 1) to identify more NIMDs patients than observer-based ratings [14], 2) to be more sensitive to sub-clinical motor changes [15], 3) to exhibit greater linearity with regard to severity [15-17], and 4) to need less training to achieve sufficient inter-rater reliability and test-retest reliability [16].

Previous studies have mostly been conducted with selected patients and non-NIMD controls, but in chronic patients using long-term antipsychotics, different NIMD often occur together [1-3].

### Studies using instrumental measurement of neuroleptic- induced akathisia (NIA)

NIA has been studied by actometry (accelerometry or actigraphy) [18-24] and by eletromyography (EMG) [25], mostly in polysomnographic studies [26,27]. For example, Gardos et al. [19] measured overall 24-hour motor activity in 8 NIA patients by means of an actometer strapped to the non-dominant ankle, and found that akathisia had no relationship to nocturnal activity. Diurnal motor activity analysis of wrist accelerometers worn by 16 NIA patients and by 16 non-NIA patients revealed that NIA patients had higher levels of motor activity over a two-day period [22]. Movement activity measured by accelerometers on ankles and the waist in a standardized rest activity (30 minutes) discriminated 10 pure NIA patients from themselves while in remission and also from 10 healthy controls with no overlap [21]. Actometry was able to reveal akathisia in a patient who had mostly subjective complaints and hypokinesia masked akathisia [23]. The same method, however, did not discriminate 31 NIA patients from those without NIA in a clinical non-selected sample of 99 patients with co-morbid NIMDs [24]. The sensitivity of actometric measurement was 0.77 and specificity 0.57 at the optimal cut-off point, which was 12000 of lower limb activity count [24].

Toe tremor was more informative than finger tremor in distinguishing 6 akathisia patients from controls by actometry. Characteristic of akathisia were low frequency (less 4 Hz) rhythmic foot movements [18]. No differences were found between NIA and pseudoakathisia (PsA) in accelerometric recordings [20]. PsA prevalence in chronic schizophrenia patients is 18% [28]. An EMG study using a case definition for akathisia (i.e. 10 second duration and less than 4 Hz in the anterior tibialis tracings) in the evaluation of 26 subjects (16 NIA patients) and yielded a sensitivity of 69% and specificity of 70% in NIA detecting [25].

### Studies of neuroleptic-induced parkinsonism (NIP)

NIP has been studied by EMG [29,30] and force transducers [14,17,31], and NIP tremor has been studied by accelerometry [32-34]. NIP tremor occurred in the range of 5–7 Hz when recorded from a pair of antagonist muscles in EMG studies, and was distinguishable from the activity of TD [29,30]. Mean tremor frequency measured by accelerometry was over 5 Hz [33]. Accelerometric recordings from 14 patients in frequencies between 4 and 7 Hz were helpful in differentiating neuroleptic-induced tremor from other NIMD and from psychogenic tremor [32].

### Studies on tardive dyskinesia (TD)

TD has been studied instrumentally by EMG [29,30], force transducers [14,16,17,31,35], and accelerometers [35-37]. EMG studies described the movement pattern as irregular bursts below 3 Hz, but almost all TD patients had activity in limbs – 15 of 16 [29,30]. Instrumental measurement has been used in TD to estimate current status and treatment efficacy [35].

Qualitative actometry (analysing the frequency peak) seemed highly specific in TD patients, with a mean Abnormal Involuntary Movement Scale (AIMS) total score of 11.2 [37].

### Aim of the study

Hyperkinetic movement disorders, like all neuropsychiatric disorders [19], may non-specifically increase rest-activity, and therefore a qualitative analysis of movement pattern may be the only way to use actometry for differentiating diagnostic purposes in naturalistic samples. The aim of our study was to analyze characteristic actometry patterns of NIMDs and PsA in a naturalistic schizophrenia population.

## Methods

We recruited 99 chronic schizophrenic institutionalized adult patients from a state nursing home in Estonia [3]. Inclusion criteria were a DSM-IV diagnosis of schizophrenia or schizoaffective disorder, stable antipsychotic medication (for at least one month), and an age of 18–65 years. Diagnosis was made using a semi-structured interview according to DSM-IV criteria for schizophrenia by a psychiatrist (SJ) and medical records. Patients with severe somatic illness or neurological illness were excluded. Written informed consent was obtained from the subjects and the study was approved by the Ethics Review Committee on Human Research of the University of Tartu.

An experienced psychiatrist (SJ) assessed all subjects to identify NIMDs (NIA, NIP, and NITD) in accordance with DSM-IV criteria [7]. PsA diagnosis was established according to Barnes and Braude [8]. NIMDs and PsA patients are in this report defined as the movement disorders group.

The temporal connection between NIMD and PsA with a neuroleptic medication was established retrospectively by interview and medical records.

Seventy nine (79.8%) patients were receiving conventional antipsychotics (mainly haloperidol, cyclopentixol, perphenazine, levomepromazine, chlorpromazine, and also thioridazine, sulpiride, chlorprotixen, fluphenazine), while 20 (20.2%) were receiving clozapine (one of whom used clozapine combined with sulpiride). No new atypical antipsychotics were used. The mean daily chlorpromazine equivalent dose [38] was 328 mg (SD 221, range 1417).

The psychiatrist (SJ) asked one subjective question from patients concerning their problems with movements: "Do you have disturbing movement problems?" The answer was allocated to one of four categories:

a) Not to my knowledge.

b) Yes, but it doesn't disturb me

c) Yes, and it disturbs me

d) Yes, and it is very difficult to cope with

The actometric recording was performed while sitting in a standardized clinical interview for 30 minutes between 9 and 11 AM, a method described previously as measuring "controlled rest activity" [21]. Controlled rest activity is a parameter of motor activity in a situation where sitting still is adequate and expected, but not instructed or required. The actometers (PAM3, IM-systems, Baltimore, USA) were attached to the ankles of the subjects to measure lower limb motor activity. Actometers are small computerized movement detectors of match-box-size which do not influence normal moving of the patient. The mode of data collection was digital integration, and the sampling rate was 40 Hz and the chosen epoch was 0.1s. PAM3 records acceleration signals exceeding 0.1G. The actometry and controlled rest activity method have been described previously by Tuisku et al. [21] and Janno et al. [24].

To test the feasibility and properties of actometric recording in a normal clinical setting we trained raters without previous experience of evaluating actometric recordings. A team of five neuropsychiatrists developed rater instructions and a data collecting form. Data evaluation training comprised two hours, followed by supervised evaluation of ten actometric recordings. Two raters (BA and AV) trained according to this procedure, and achieved an appropriate level of inter-rater reliability (0.44 to 1.0, mean 0.82) during their training phase. Raters were blinded and had no access to patient data other than the actometric recording.

The two raters evaluated all study subjects' actometric activity recordings for the existence of activity periods, the duration of activity periods (activity for at least 10 seconds), the existence of rhythmical activity. Raters calculated from persistent rhythmical activities three most dominant frequencies for every patient (if patients had rhythmical activity with different frequencies). Raters found the highest acceleration peaks in activity periods in the scope of 10 seconds. After calculating inter-rater reliability, a meeting between raters was held to establish consensus values for the estimated frequencies of activity periods for 27 patients. The extracted data was then assessed by KT and SJ to find any patterns for individual NIMDs and PsA. Answers to the subjective question were analyzed for a correlation with movement disorders diagnoses.

The inter-rater reliability was measured by kappa coefficients for categorical values and intra-class correlation (ICC) coefficients for continuous values. A two-way ANOVA mixed model was used to calculate ICC, so as to estimate the reliability of a single rating [39]. One-way ANOVA was performed to analyze the ability to discriminate different qualities of movement patterns. Differences between the movement disorder (NIA, NIP, TD and PsA) and the non-movement disorder groups in activity periods were analyzed by the Mann-Whitney two-tailed U-test for continuous variables (frequency, amount of periods). Chi-square analyze was used for dichotomous variables (presence of activity periods, rhythmical activity). Where necessary, Fisher's exact test was used for calculating significance. The performances of movement patterns in case identification were evaluated by receiver operating characteristics (ROC) analyses. The software used in analyses was SPSS 12.0 (SPSS Inc. Chicago, Illinois, USA).

## Results

A total of 88 patients (89%) showed rhythmical activity and their results were used in the analysis. Raters achieved excellent inter-rater reliability in almost all parameters of movement patterns of actometric recordings, except defining the minimal frequency of movement patterns (Table 1).

**Table 1:** Inter-rater reliability coefficients for actometry variables in 99 in-patients with schizophrenia: kappa for categorical and ICC coefficients for continuous measures.

	**kappa**
presence of activity periods	0.905*
presence of rhythmical activity	0.786*
	**ICC **(95% CI)
amount of activity periods	0.967 (0.951–0.978)*
dominant frequency	0.739 (0.624–0.822)*
second prevalent frequency	0.787 (0.672–0.864)*
third prevalent frequency	0.789 (0.672–0.867)*
minimal frequency	0.351 (0.149–0.525)#
maximal frequency	0.831 (0.750–0.887)*
highest value of acceleration peaks	0.841 (0.768–0.893)*

* p < 0.001

# p = 0.001

Movement disorders group patients had more activity periods in actometric recordings than other patients (Table 2). More than 95% of NIA and PsA patients showed rhythmical activity. Patients who had movement disorder and particularly NIP, PsA and NIA patients had higher frequencies in rhythmical activity than other patients (Table 3). NIMD and PsA, except TD, patients had higher median acceleration peaks in rhythmical activity recordings than non-movement disorders group patients (U = 0.575, p = 0.024).

**Table 2:** Presence of activity periods and rhythmical activity in differentiating neuroleptic-induced movement disorders for 99 in-patients with schizophrenia.

	Presence of activity periods	Presence of rhythmical activity
**non-movement disorders vs.** **NIMD and PsA**		
Pearson chi-square	5.547	2.726
p	0.035	0.099
**NIA vs. non-NIA**		
Pearson chi-square	5.642	5.574
p	0.016	0.019
**NIP vs. non-NIP**		
Pearson chi-square	0.113	4.505
p	0.714	0.034
**TD vs. non-TD**		
Pearson chi-square	1.131	1.607
p	0.495	0.254
**PsA vs. non PsA**		
Pearson chi-square	0.814	2.061
p	0.685	0.151
**Pooled NIA and PsA vs. no akathisia**		
Pearson chi-square	8.491	11.027
p	0.004	0.001

**Table 3:** Neuroleptic-induced movement disorders patterns for 99 in-patients with schizophrenia.

	Presence of activity periods n%	Median amount of activity periods IQ*	Presence of rhythmical activity n%	Median of dominant frequency Hz IQ*	Median of second frequency Hz IQ*	Median of third frequency Hz IQ*	Median of minimal frequency Hz IQ*	Median of maximal frequency Hz IQ*	Median highest value of acceleration peaks IQ*
non-movement disorders n = 32	25 78%	2 1.00–7.63	24 75%	0.50 0.40–0.59	0.43 0.40–0.61	0.55 0.48–0.78	0.33 0.30–0.45	0.65 0.50–0.98	106 57–193
NIA n = 31	31 100%	13 5.00–22.00	30 97%	0.58 0.40–1.01	0.60 0.45–0.80	0.70 0.49–1.01	0.38 0.30–0.45	1.15 0.74–2.18	177 81–226
NIP n = 23	20 87%	8 1.00–14.50	16 70%	0.75 0.50–0.95	0.73 0.51–4.11	0.93 0.60–1.09	0.40 0.35–0.49	1.05 0.65–2.26	216 140–250
TD n = 32	30 94%	9.75 3.00–16.75	29 91%	0.55 0.40–0.80	0.55 0.45–0.93	0.65 0.46–0.95	0.40 0.30–0.45	0.95 0.63–2.03	125 75–206
PsA n = 19	18 95%	16 10.50–25.50	18 95%	0.80 0.50–1.53	0.80 0.49–1.00	0.60 0.49–1.04	0.40 0.30–0.43	1.15 0.75–2.58	127 79–208

All patients n = 99	88 89%	8 2.00–17.50	83 84%	0.55 0.40–0.80	0.55 0.40–0.80	0.65 0.50–0.98	0.35 0.30–0.45	0.90 0.60–1.45	129 80–213

* Interquartile range

The presence of rhythmical activity, amount of activity periods, maximal frequency, and highest values of acceleration peaks differentiated between the subgroups of NIMDs and PsA. The other parameters of movement patterns showed no ability to discriminate between movement disorders groups by one-way ANOVA analysis (data not shown). Presence of activity periods and rhythmical activity in actometric recordings had the best abilities to discriminate the patient group of pooled NIA and PsA (Table 2).

The PsA group differed mostly from the non-movement disorders group in regard to median number of activity periods and frequencies. The results of particular NIMD groups and non-movement disorders group are presented in Table 4.

**Table 4:** The differences in median values of actometric pattern qualities of particular neuroleptic induced movement disorders from non-movement disorder group for 99 in-patients with schizophrenia

	amount of activity periods	dominant frequency	second frequency	maximal frequency	median maximum amplitude
NIA median	13	0.58	0.60	1.15	177
non-NIMD median	2	0.50	0.43	0.65	106
Mann-Whitney U	207	262	153	177	277
p	<0.001	0.084	0.017	0.001	0.040
NIP median	8	0.75	0.73	1.05	216
non-NIMD median	2	0.50	0.43	0.65	106
Mann-Whitney U	282	127	58	143	145
p	0.140	0.040	0.009	0.106	0.023
TD median	9.75	0.55	0.55	0.95	127
non-NIMD median	2	0.50	0.43	0.65	106
Mann-Whitney U	294	267	163	209	305
p	0.003	0.145	0.030	0.013	0.110
PsA median	16	0.8	0.8	1.15	144
non-NIMD median	2	0.50	0.43	0.65	106
Mann-Whitney U	87	103	82	92	156
p	< 0.001	0.004	0.011	0.002	0.042
NIA and PsA median	15.75	0.68	0.60	1.15	155
non-NIMD median	2	0.50	0.43	0.65	106
Mann-Whitney U	641	364	235	269	433
p	0.003	0.011	0.006	< 0.001	0.017

The differences between particular movement disorder and non-movement disorder groups in the highest acceleration peak medians are presented in Table 4. Highest acceleration peak medians (i.e. greatest digital integration of acceleration) in activity periods were almost significantly (p = 0.053) different (Mann-Whitney U = 444) between NIP (203, interquartile range (IQ) 89, 250) and non-NIP (116, IQ 67, 190) groups. No significant differences were found between other groups.

Median values of third frequency and minimal frequency were not statistically significantly different between groups.

To evaluate screening properties of actometry against DSM-IV and PsA diagnostic criteria we used ROC analysis. The area under the ROC curve (AUC) of the lower limb activity count was 0.80 for PsA and 0.84 for pooled akathisia (NIA and PsA).

**Figure 1:**
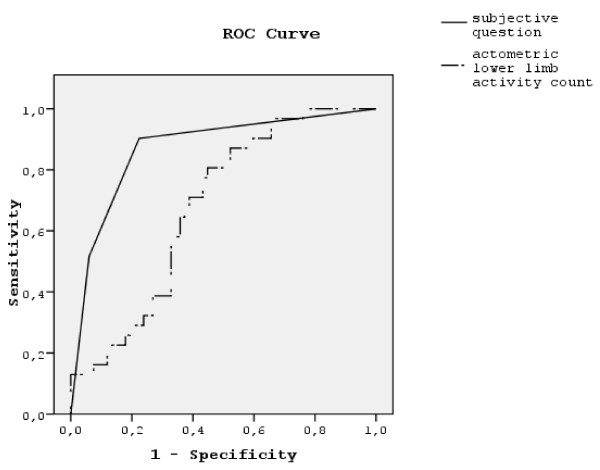
Receiver Operating Characteristic (ROC) curves for subjective question and lower limb activity count against DSM-IV defined neuroleptic-induced akathisia (NIA).

Answers to the subjective question differed between the movement disorders and the non-movement disorder group (Pearson chi-square 8.209, p = 0.004). The ROC curve for the screening performance of the subjective question in NIA is presented in Figure 1. ROC analysis of the subjective question for differentiating abilities showed an AUC value of 0.67 for NIMD and PsA, 0.87 for NIA, 0.52 for NIP, 0.46 for TD, and 0.25 for PsA.

Figure 1. Receiver Operating Characteristic (ROC) curves for subjective question and lower limb activity count against DSM-IV defined neuroleptic-induced akathisia (NIA).

## Discussion

Our main finding was that actometry could objectively differentiate NIA and PsA patients from the non-movement disorder group and from all the other NIMD patients. Also, the subjective question on movement problems was able to detect NIA patients from all other subjects. Answers to this question were highly selective for NIA cases, but not for other NIMDs and PsA.

The strength of the study is the naturalistic non-selected patient sample. Therefore, we can generalize results to other chronic schizophrenia in-patients. It is possible that co-morbid movement disorders, existing in this real-life sample, compromise the previously reported characteristics of individual NIMDs or PsA. Therefore, a loss of differentiating ability of actometry is seen in a clinical setting like ours.

A limitation of the study is that for practical reasons, the clinical diagnosis of NIMD or PsA was made and the subjective question was asked by the same clinician, which could have contaminated the investigator's judgement.

Another limitation of our study was that it could not detect the sub-clinical movement disorders, because we used the DSM-IV [7] and PsA [8,20] diagnostic criteria as the "gold standard": These criteria do not identify sub-clinical cases. Thus, the non-movement disorder subgroup may include sub-clinical movement disorder subjects, who were not detectable by DSM-IV criteria for NIMD. The latter is supported by the following findings: 1) even 75% of non-movement disorder subjects showed rhythmical activity which can be related to movement disorders, seldom a normal activity during rest; and 2) the non-movement disorder patients had less activity periods in recordings, but when they had, then frequencies were quite similar to NIMD or PsA (mostly TD) subjects.

### NIA and PsA

In this sample, we have previously shown that quantitative actometry does not discriminate NIA patients from those without NIA [24]. Our finding contrasted with the finding of Tuisku et al. [21], who reported a good discrimination by actometry of selected NIA patients and healthy controls. It seems that the inclusion of PsA (which is a hyperactive movement disorder and similar in actometric recordings to NIA) in the non-NIA group, in accordance with DSM-IV criteria, compromised the differentiating ability of actometry in this population [24].

Our findings are consistent with a previous accelerometric study [20], which found no differences between clinical observations and accelerometric recordings of NIA and PsA. We have previously reported that in NIA the area under the ROC curve was 0.68 [24] for the actometric activity count, and we now report that in PsA the discriminatory power of actometry is better (ROC 0.80). The overall activity count differentiated the pooled NIA and PsA from other NIMD and non-movement disorder patients.

Differences in median values of the actometric count for the PsA group and non-movement disorder group validates the diagnosis of PsA. These patients have higher frequency movements than other patients, but are not aware of them (according to answers to our subjective question). They have difficulties in noticing their disturbance, which clearly distinguishes this disorder from NIA.

The subjective question was selective for NIA cases, but not for other NIMDs or PsA. This is explained by the subjective component of NIA. Asking such a subjective question offers a cost-effective way of screening NIA with good screening ability. Although the single question has its limitations and is not enough to evaluate the various subjective discomforts associated with NIMD. Our results show that even single question can be a useful method of diagnosing NIA in a clinical setting.

### NIP

The parkinsonian tremor shows rhythmical activity in accelerometric recordings, which discriminates NIP patients from non-movement disorder patients. NIP patients had higher peaks of accelerations in activity periods than other patients. Comparing the NIP group with the non-movement disorder group made this finding more significant. Parkinsonian hypokinesia, unlike other NIMD or PsA, shows less movements (overall activity count) in actometric recordings, which we have presented previously [34]. In our study, the frequencies of rhythmic activity in NIP were much lower (median below 1 Hz), than reported by previous studies (above 4 Hz) [29,33]. One possible explanation is that by measuring lower limb activity in a sitting position we could not detect the high frequency NIP tremor, which in previous studies was measured from upper limbs by actometry [33]. There may have been a high frequency tremor (close to 10 Hz) in our sample that we were not able to detect with our method due to the limitation of the time window of our actometers.

### TD

TD usually affects only some body segments, mostly the orofacial region and sometimes the legs. Recording of ankle movements gave little information for detecting TD, despite the fact that the movement pattern of TD has more activity periods, rhythmical activity, and somewhat higher frequencies than non-movement disorder patterns.

## Conclusion

The actometric recording and analysing of the standardized left-leg motor activity is moderately time-consuming, but it may have limited use. The method can be used in NIMD and PsA patients for differentiating akathisia (pooled NIA and PsA) from other patients, but not for discriminating between NIA and PsA. Our study employed ankle recording only, and actometric recording from the ankle and wrist simultaneously may give more information for detecting NIMD in the schizophrenia patient population. Actometry allows a quantifiable recording of current state of movement disorders and is thus useful in research settings. Further research should focus on the ability of actometry to show change in the patient condition. Initial studies in this direction have already been done [21,35].

Actometry is useful for measuring change in the overall movement count or patterns after a change of risk factors (dosage, antipsychotic type, time course etc) in experimental conditions, and further research can show the ability of actometric data to drive treatment decisions in a clinical setting.

The present study showed that pooled NIA and PsA patients had different patterns in lower limb descriptive actometry than other patients in a non-selected sample. Careful questioning of patients is a useful and clinically significant method of diagnosing NIA in a clinical setting.

## Competing interests

The author(s) declare that they have no competing interests.

## Authors' contributions

SJ contributed to the study design, collected the data, contributed to the analyses and data interpretation, made the literature search and was responsible for manuscript preparation. MMH contributed to study design, analyses, data interpretation, and manuscript preparation. KT contributed to study design, statistical analysis, data interpretation and manuscript preparation. KW supervised the study design and contributed to statistical analysis, data interpretation and preparation of the manuscript.

## Pre-publication history

The pre-publication history for this paper can be accessed here:


